# A Bio-mechanical Model for Elbow Isokinetic and Isotonic Flexions

**DOI:** 10.1038/s41598-017-09071-x

**Published:** 2017-08-21

**Authors:** Xi Wang, Xiaoming Tao, Raymond C. H. So

**Affiliations:** 1 Institute of Textiles and Clothing, The Hong Kong Polytechnic University, Hong Kong, China; 2 Multidisciplinary Division of Bioengineering, The Hong Kong Polytechnic University, Hong Kong, China; 3 Hong Kong Sports Institute, Hong Kong, China

## Abstract

A new bio-mechanical model for elbow flexions is proposed to quantify the elbow torque generated as a function of the upper-arm circumferential strain and influencing factors of elbow angle and angular velocity. The upper-arm circumferential strain is used to represent the contractile intensity of the dominant flexor, biceps brachii, whose behavior is described by Hill’s theory. Experiments with thirteen healthy subjects were conducted to determine the influencing factors. The temporal distributions of torque and elbow angle were measured by Biodex ®3 simultaneously, while the upper-arm circumference was obtained by a wearable anthropometric measurement device. Within the experimental range, the change of angular velocity has been found to have no effect on the torque generated. The new model was further verified experimentally with reasonable agreements obtained. The mean relative error of the torque estimated from the model is 15% and 22%, for isokinetic and isotonic flexions, respectively. The verified model establishes the relationship between the torque generated and circumference strain of the upper arm, for the first time, thus provide a scientific foundation for the anthropometric measurement technology as an alternative to sEMG for monitoring force/torque generation during elbow flexions.

## Introduction

Fundamental studies of skeletal muscles and their nerve control in human activities have led to the successful development of a range of measurement technologies which have been applied for diagnosis of muscle and nerve related diseases, monitoring of health conditions and activities in sports, prevention of muscle fatigue and injury, as well as rehabilitation. Since Hill^1^ revealed the bio-mechanism of muscle contraction and proposed three-element model of skeletal muscle, the two fundamental relationships of skeletal muscles, i.e., the force-length and force-velocity behavior^1^, have been widely studied and incorporated into a variety of bio-mechanical models. Bigland and Lippold^2^ incorporated surface bio-potentials, indicating that the contractile force of skeletal muscle can be determined by the maximum force (MVC), the activation level, force-length factor and force-velocity factor^3^. The activation level of skeletal muscles is constructed from surface electromyography (sEMG) signals, while the maximum force is normally obtained by calibration. The influencing factors of muscle length and shortening velocity, however, can be found in diverse forms with tuning parameters, specifically applicable to different skeletal muscles^3–5^. Zajac^6^ and Winters^3^ improved the earlier models by introducing the effect of the pennation angle of skeletal muscles. These sEMG-driven models have been adapted for biceps brachii and proved effective for emulating nerval controls, contractions as well as other muscular behaviors^7, 8^. Despite the successful applications of the sEMG-driven models in neurophysiology and biomechanics, the sEMG devices are intricate, susceptible to variations in moisture and positions of electrodes, thus are used in well-conditioned laboratories instead of fields.

Apart from sEMG technology, morphological parameters of skeletal muscles, such as fascicle length, pennation angle and cross-sectional area (CSA) or muscle thickness, have also been reported as indexes of muscle contraction in both static and dynamic conditions, using imaging methods^9–12^. Decreasing fascicle length and increasing fiber pennation angle of skeletal muscles have been observed during contraction^9, 10, 12^, induced by shortening of muscle fibers in length and expansion in diameter. In particular, cross-sectional area and thickness of biceps brachii were observed increasing during isometric contraction^11, 13–16^. Hodges^17^ reported that the muscle thickness increased with the amplitude of sEMG in a negative exponential style, i.e., almost linearly in low-contraction (<30% MVC) then much slower in high-contraction. Similar findings were reported by other groups^14, 18–20^, establishing muscle thickness as additional index of muscle contraction.

Varied by thickness of skeletal muscle within, limb circumferences have recently been studied through anthropometric measurements and proved another index of muscle contraction^13, 16, 21^. Contraction of biceps brachii has been specially studied with upper-arm circumferences^22, 23^. In a field study with a lab-made wearable smart sensing device, namely the limb gauge measurement system (LGMS), the authors’ group found exponential correlations between the sEMG RMS and upper-arm circumference, as well as linear relationships between the upper-arm circumferential strain and joint torque during elbow isometric contractions^24^. Furthermore, the slope of the linear correlation was observed to decline as the elbow angle increases^24^, demonstrating a significant effect of muscle length.

Compared to sEMG and imaging technology, wearable anthropometric measurement is a novel but more convenient way to keep long-term tracking of muscle contraction, potentially applicable for both laboratories and fields. However, most related works were conducted in isometric mode, instead of kinetic^13, 16, 21–24^. The effect of muscle length has not been confirmed in kinetic flexions and the effect of shortening velocity on biceps’ contraction has never been addressed. Moreover, no theoretical treatment in literature has related the torque generation, during elbow flexion, with anthropometric measurements. This research gap has hindered the development of anthropometric measurement technologies as alternatives of the widely used sEMG.

Hence in this paper, a new bio-mechanical model of elbow flexion will be developed, inspired by Hill’s descriptions of skeletal muscles. Torque during elbow flexions will be related to upper-arm circumference strain and influencing factors. The effects of the elbow angle (corresponds to effects of the length of biceps brachii) and angular velocity (corresponds to effects of the shortening velocity) will be addressed and determined. Furthermore, the model will be experimentally verified in both isokinetic and isotonic modes. Error analysis will be conducted, followed by discussions and conclusions.

## Methods

### Hypothesis and modeling

**Figure 1:**
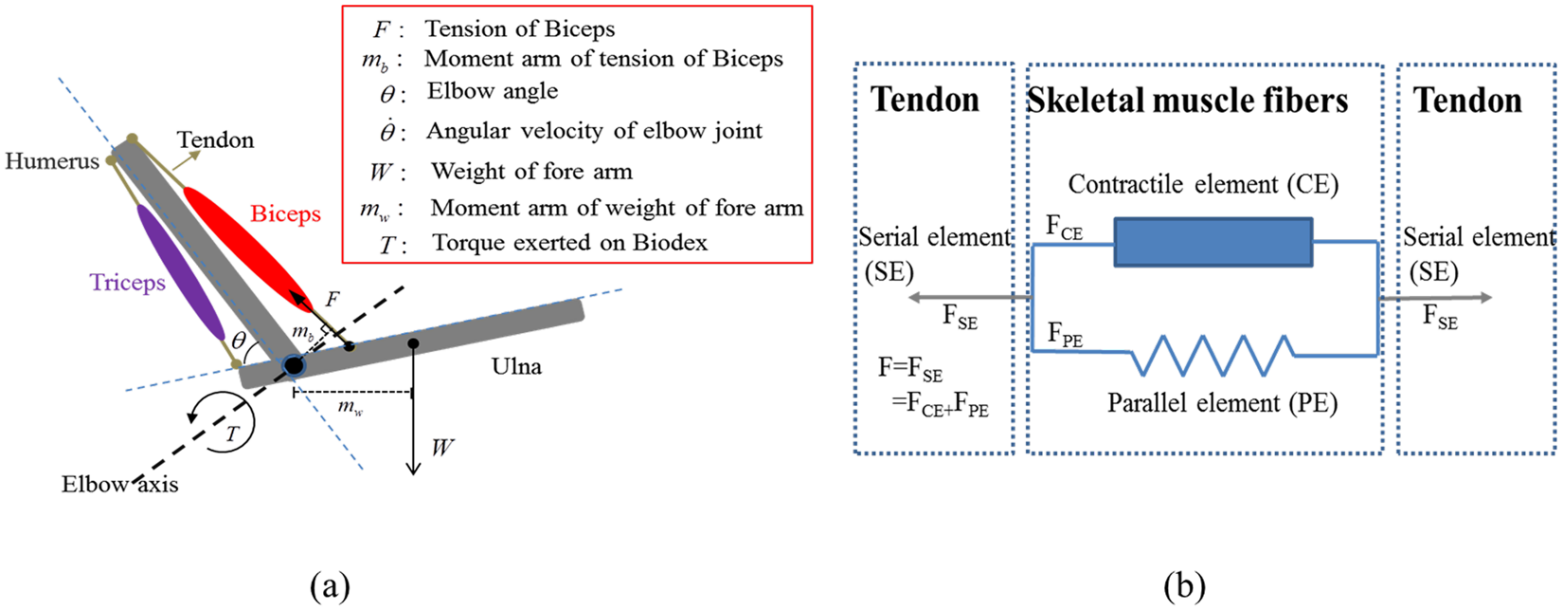
(**a**) Muscular-skeleton model of human elbow. (**b**) 3-elements of a typical skeletal muscle according to Hill^1^.

As shown in Fig. 1(a), the elbow joint is treated as a bio-mechanical system comprising two antagonistic muscles, biceps brachii (dominant elbow flexor) and triceps brachii (dominant elbow extensor), as well as two skeleton bones, humerus and ulna. The elbow angle *θ* is the angle between humerus and short end of ulna, $\dot{\theta }$ denotes the angular velocity of flexion, *F* is the contractile force of the biceps brachii and *W* is the weight of the fore-arm. The moment arm of *F* and *W* are *m*_*b*_ and *m*_*W*_ respectively. An external torque, *T*, is exerted on the system.

The overall external torque exerted on the elbow joint (*T*_*all*_) is equal to the summation of the torques generated by the biceps brachii (*T*_*b*_), triceps brachii (*T*_*t*_) and the weight of forearm (*T*_*W*_):

1 $${T}_{all}={T}_{b}+{T}_{t}+{T}_{W}$$

Voluntary elbow flexions are focused in the present work. An assumption is made that the triceps brachii does not contribute co-contractions in maximum flexions^25, 26^, the tension force and torque generated by triceps brachii (*T*_*t*_) are neglected and the above equation becomes

2a $${T}_{all}={T}_{b}+{T}_{W}$$

where *T*_*b*_ = *F* ⋅ *m*_*b*_ and *T*_*W*_ = *W* ⋅ *m*_*W*_.

According to Hill’s theory^1, 27, 28^, the biceps brachii can be treated as a combination of serial element (SE), contractile element (CE) and parallel element (PE), as shown in Fig. 1(b). The total contractile force of the biceps brachii is the summation of the contractile force of CE (*F*_*CE*_) and the passive stretch force of PE (*F*_*PE*_).

2b $$F={F}_{CE}+{F}_{PE}$$

where *F*_*CE*_ has been described as a function involving four factors, that is, the maximum voluntary contractile force (MVC, denoted by *F*_*m*_), the activation level (*α*), the force-length factor (*g*_*l*_) and the force-velocity factor (*g*_*v*_), as in the following equation

2c $${F}_{CE}={F}_{m}\cdot \alpha \cdot {g}_{l}\cdot {g}_{v}$$

The tension of passive element, *F*_*PE*_, has been treated as a nonlinear spring. It can be considered negligible over most of the motion range and with a peak of less than 10% of *F*_*m*_ at the maximum anatomical length^29^. With the humerus fixed in this study, *m*_*W*_ is a function of elbow angle *θ*, the torque by weight $W\cdot {m}_{W}$ can be eliminated through self-weight calibration (as provided by Biodex®3). Thus equation (2b) becomes:

3 $$T={T}_{all}-W\cdot {m}_{W}={F}_{CE}\cdot {m}_{b}$$

After the self-weight calibration, the measured torque *T* is actually exclusively contributed by *F*_*CE*_ alone. Substitute *F*_*CE*_ in equation (2c) into equation (3) yields

4 $$T={T}_{m}\cdot \alpha \cdot (\frac{{F}_{m}}{{F}_{op}}\cdot \frac{{m}_{b}(\theta )}{{m}_{op}}\cdot {g}_{l}(\theta ))\cdot {g}_{v}(\dot{\theta })$$

where *T*_*m*_ = *F*_*op*_ ⋅ *m*_*op*_, *F*_*op*_ and *m*_*op*_ are the contractile force and moment arm when maximum torque *T*_*m*_ is generated. The above equation shows that in the elbow muscular-skeleton system, the generated torque in flexions depends on the activation, the elbow angle (*θ*, representing the length of biceps brachii) and elbow angular velocity ($\dot{\theta }$, representing the shortening velocity of biceps brachii). With the angle-related constants merged for simplicity, the above equation can be re-written as

5 $$T={T}_{m}\cdot \alpha \cdot {\tilde{g}}_{l}(\theta )\cdot {g}_{v}(\dot{\theta })$$

where $${\tilde{g}}_{l}\,=\frac{{F}_{m}}{{F}_{op}}\cdot \frac{{m}_{b}(\theta )}{{m}_{op}}\cdot {g}_{l}(\theta )$$

The activation level *α*, which represents the activation level of muscle units bio-electrically, is commonly constructed from sEMG rms by fitting to specific activation-force patterns for various skeletal muscles^8, 30^.

It has been reported that normalized sEMG rms of biceps is correlated with upper-arm circumferential strain and normalized muscle thickness in isometric flexions^14, 20, 24^. This indicates that, the activation level (*α*) can be expressed as a function of the circumferential strain (*s*) for isometric flexions. Furthermore, the elbow torque and circumferential strain demonstrated a good linear relationship during isometric flexions^24^, when *g*_*l*_ and *g*_*v*_ were reduced to constants (the elbow angle was kept fixed and angular velocity was zero). In equation (5), it is reasonable to assume an approximation of activation level *α* by truncating its Taylor expansion to the first order:

6 $$\alpha =\alpha (s)\approx k\cdot s+b$$

where *b* = 0, since no circumferential change will happen when the biceps brachii is totally relaxed. Equation (6) is applicable for isometric flexions only, with the slope *k* depending on current elbow angle (*θ*)^24^. An extension of equation (6) is assumed for general flexions, by introducing a factor of angular velocity (*k*_2_) in the slope (*k*):

7a $$k={k}_{1}(\theta )\cdot {k}_{2}(\dot{\theta })$$

where *k*_1_ and *k*_2_ are exclusively effected by elbow angle (*θ*) and angular velocity ($\dot{\theta }$), respectively. Substitute equation (6) and (7a) to equation (5),

7b $$T=s\cdot {T}_{m}\cdot {G}_{l}(\theta )\cdot {G}_{v}(\dot{\theta })$$

where ${G}_{l}(={k}_{1}(\theta )\cdot {\tilde{g}}_{l}(\theta ))$ is the overall torque-angle factor (corresponding to the force-length factor in Hill-based models) and ${G}_{v}(={k}_{2}(\dot{\theta })\cdot {g}_{v}(\dot{\theta }))$ is the overall torque-velocity factor (overall effect of elbow angular velocity ($\dot{\theta }$), corresponding to the force-velocity factor in Hill-based models), which will be examined experimentally later in kinetic flexions, including the isokinetic flexions and isotonic flexions. Equation (7b) describes a new bio-mechanical model of the elbow flexions driven by circumferential measurements. For ordinary people, circumferential strain *s* varies within 0~30%^24^. If a normalized circumferential strain *β* (0 ≤ *β* ≤ 1) is further utilized,

7c $$\beta =\frac{s}{{s}_{\max }}$$

where ${s}_{\max }(\theta )$ is the maximum circumferential strain obtained in isometric MVC flexions at elbow angle *θ*. The proposed model can be re-arranged as

7d $$T={T}_{m}\cdot \beta \cdot {\hat{G}}_{l}(\theta )\cdot {\hat{G}}_{v}(\dot{\theta })$$

where ${\hat{G}}_{l}(={k}_{1}(\theta )\cdot {s}_{\max }(\theta )\cdot \frac{{F}_{m}}{{F}_{op}}\cdot \frac{{m}_{b}(\theta )}{{m}_{op}}\cdot {g}_{l}(\theta ))$ and ${\hat{G}}_{v}(={k}_{2}(\dot{\theta })\cdot {g}_{v}(\dot{\theta })))$. In this case *β* may play a similar role as ‘activation’.

### Experimental setup

The setup of the experiment is the same as in previous work^24^. Lab-made LGMS, Biodex ^®^3 (Biodex isokinetic testing system 3, New York, USA) and its elbow attachments were used. The LGMS’s sensing belt was mounted in specified location on subject’s upper arm to measure the upper-arm circumference^24^. Each subject was equipped with selected size of sensing belt to ensure the fabric sensors of LGMS working in effective range. The range of motion (ROM) were configured on the user interface of Biodex 3 and set identically for all subjects as 30°~120°. In this work, the protocols involved only cyclic unilateral elbow flexion-extension movements in kinetic modes (isokinetic and isotonic flexions).

### Calibration of connatural circumference and the circumferential strain

Both voluntary contraction and passive shortening increase the thickness of biceps brachii and eventually raise the upper-arm circumference. To eliminate the effect of the passive shortening of biceps brachii, for kinetic flexions, the connatural circumference within the ROM should be acquired first through a calibration process: during passive isokinetic flexions (subjects flexed elbow with the help of lab assistant and no voluntary tension generated by elbow flexors), upper-arm circumferences of each subject were measured and recorded by the LGMS as a function of elbow angle. Circumferential strain *s* is defined to extract the circumferential change due to voluntary contraction only,

7e $$s=\frac{C(\theta )-{C}_{0}(\theta )}{{C}_{0}(\theta )},\,\forall \theta \in {\rm{ROM}}$$

where *C* is measured circumference, while *C*_0_ is calibrated connatural circumference.

In this work, isokinetic flexions were used to separate and determine the factor of angular velocity and factor of elbow angle in the new model. Afterwards, verification of the model would be conducted for both isokinetic and isotonic flexions.

### Isokinetic test

Three angular velocities were chosen for the isokinetic contraction tests with the Biodex system, 60°/s, 90°/s and 120°/s. Each subject took two sets of action under each speed, each action comprises four consecutive cycles of extension-flexion, i.e., two sets for 90°/s, two sets for 120°/s and two sets for 60°/s, in time-sequence. Subjects were encouraged to make their maximum efforts for each flexion only, and rest during extensions. One-minute breaks were set between adjacent attempts to avoid fatigue. Temporal joint torque and elbow angle were recorded by the Biodex® 3, while the real-time upper arm circumferences were recorded by the LGMS. An impulse circuit synchronized the three signals.

### Isotonic test

Two levels of impedance were selected for each subject. Since the strength of biceps brachii varies among subjects, the impedance of flexion provided by Biodex was set to approximate 30% and 40% of the maximum torque produced by the subject in the isokinetic test at 120°/s (recorded as T_120_). For instance, the maximum torque of subject No.3 is about 32 N•m in his isokinetic test at 120°/s, hence, the impedance was set to 10 N•m and 15 N•m for his own isotonic tests. The reasons for the chosen impedance levels instead of MVC or higher are: firstly, to make sure subjects can perform full flexions within the ROM, and secondly, to avoid muscle fatigue in biceps brachii. Each subject took four sets of actions, i.e., two for the 30% T_120_ impedance and two for 40% T_120_ impedance. The subjects were encouraged to try their maximum contraction for each flexion only. One minute breaks for rest were set between adjacent sets. Devices and measurements for joint torque, elbow angle and upper arm circumferences were identical to those used in the isokinetic tests.

### Subjects

Fifteen volunteer were enrolled, in which thirteen healthy right-handed male subjects with diverse habits of sports were selected (as in Supplementary Table S1). Subjects with a maximum circumferential strain *s* of less than 5% during flexions were excluded to avoid the invalid working range of the LGMS. The Human Subjects Ethics Sub-committee of The Hong Kong Polytechnic University reviewed and approved the research plan. All subjects were informed of data privacy and testing risks before test, and signed consents were obtained. All methods were performed in accordance with the relevant guidelines and regulations stipulated by the University.

### Data availability

The data that support the findings of this study are available from the corresponding author on reasonable request.

### Data analysis

#### Rectifying circumference

Resistance relaxation of the fabric strain sensors in the LGMS has been reported at fixed strain, along with mechanical relaxation of the elastic fabrics^31, 32^. To avoid the error caused by the drift of resistance due to relaxation in longtime test (about 20~30 min for each subject), the profile of the measured circumference will be analyzed instead of the absolute value, through a rectification process.

7f $$\mathop{c}\limits^{\frown {}}=\frac{C(\theta )-C(30^\circ )}{C(120^\circ )-C(30^\circ )}\cdot (\bar{C}(120^\circ )-{C}_{0}(30^\circ ))+{C}_{0}(30^\circ )$$

where *C*(*θ*) is the measured circumference within ROM, *C*_0_(30°) is the circumference at 30° while *C*_0_(120°) is the circumference at 120°, $\bar{C}(120^\circ )$ is the averaged circumference at transitions from flexions to extensions. This rectification eliminated the error caused by the base value drift of LGMS, through fitting all the obtained circumferences in flexions to a fixed frame. Validity of the rectification was automatically supported by the testing protocols. First, any flexion should start from relaxation (with relaxed biceps brachii). Hence, the circumferences measured at the initial of the ROM (30°) were of the same value (*C*_0_(30°)). Secondly, any flexion should end by a transition of flexion-to-extension, when the joint torque decreased to zero and then negative. Therefore, the circumferences measured at the end of the ROM, when the torque was zero, were of another same value ($\bar{C}(120^\circ )$). Therefore, the circumferential strain due to voluntary contraction of biceps brachii can be extracted as

8 $$s=\frac{\mathop{c}\limits^{\frown {}}(\theta )-{C}_{0}(\theta )}{{C}_{0}(\theta )}$$

### Combined factor of torque-angle and torque-velocity properties

For the convenience of analysis, a simpler form of equation (7b) will be implemented

9 $$T=s\cdot {\mathop{G}\limits^{\frown {}}}_{l}\cdot {G}_{v}$$

where ${\mathop{G}\limits^{\frown {}}}_{l}\,(={T}_{m}\cdot {G}_{l}(\theta ))$ is an overall torque-angle factor (unit: N·m). Move the strain *s* to the left, we have the combined factor of angle and velocity, *η*,

10 $$\eta =\frac{T}{s}={\mathop{G}\limits^{\frown {}}}_{l}(\theta )\cdot {G}_{v}(\dot{\theta })$$

where *η* is the production of torque-angle factor ${\mathop{G}\limits^{\frown {}}}_{l}(\theta )$ and torque-velocity factor ${G}_{v}(\dot{\theta })$. Since the circumferential strain *s* approaches 0 at both limits of the ROM, the combined factor *η* would be much greater at limits of the ROM than that within the ROM. Then $\mathrm{lg}\,\eta$ is analyzed instead for the impact of elbow angle and angular velocity.

## Results

### The torque-velocity factor (Effect of angular velocity)

**Figure 2:**
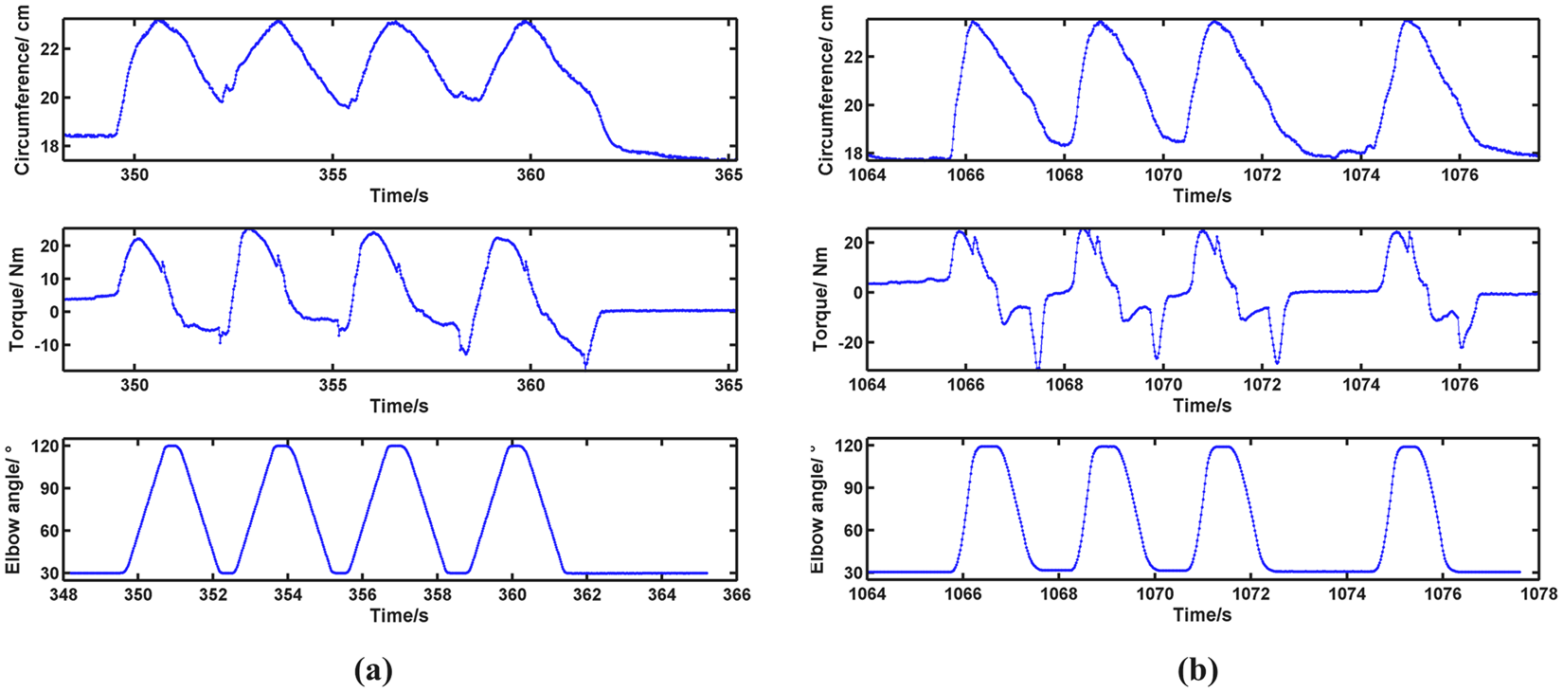
(**a**) Raw data of circumference, torque and elbow angle in isokinetic test (Subject No. 1’s 1^st^ isokinetic set at 90°/s). (**b**) Raw data of circumference, torque and elbow angle in isokinetic test (Subject No. 1’s 1^st^ isotonic set at 30% T_120_).

Totally thirteen data sets of joint torque, elbow angle and upper-arm circumference in the isokinetic tests were jointly analyzed. Of all the four cycles of flexion-extension in one isokinetic attempt, only the 2^nd^ flexion was chosen for analysis. The reasons are, firstly, the sensing behavior of fabric strain sensors in LGMS was repeatable except for the 1^st^ cycle^31, 32^; secondly, to reduce error caused by resistance relaxation of the fabric sensors, the 2^nd^ cycle was with less error caused by resistance relaxation other than the 3^rd^ and 4^th^. Typical raw data are shown in Fig. 2.

**Figure 3:**
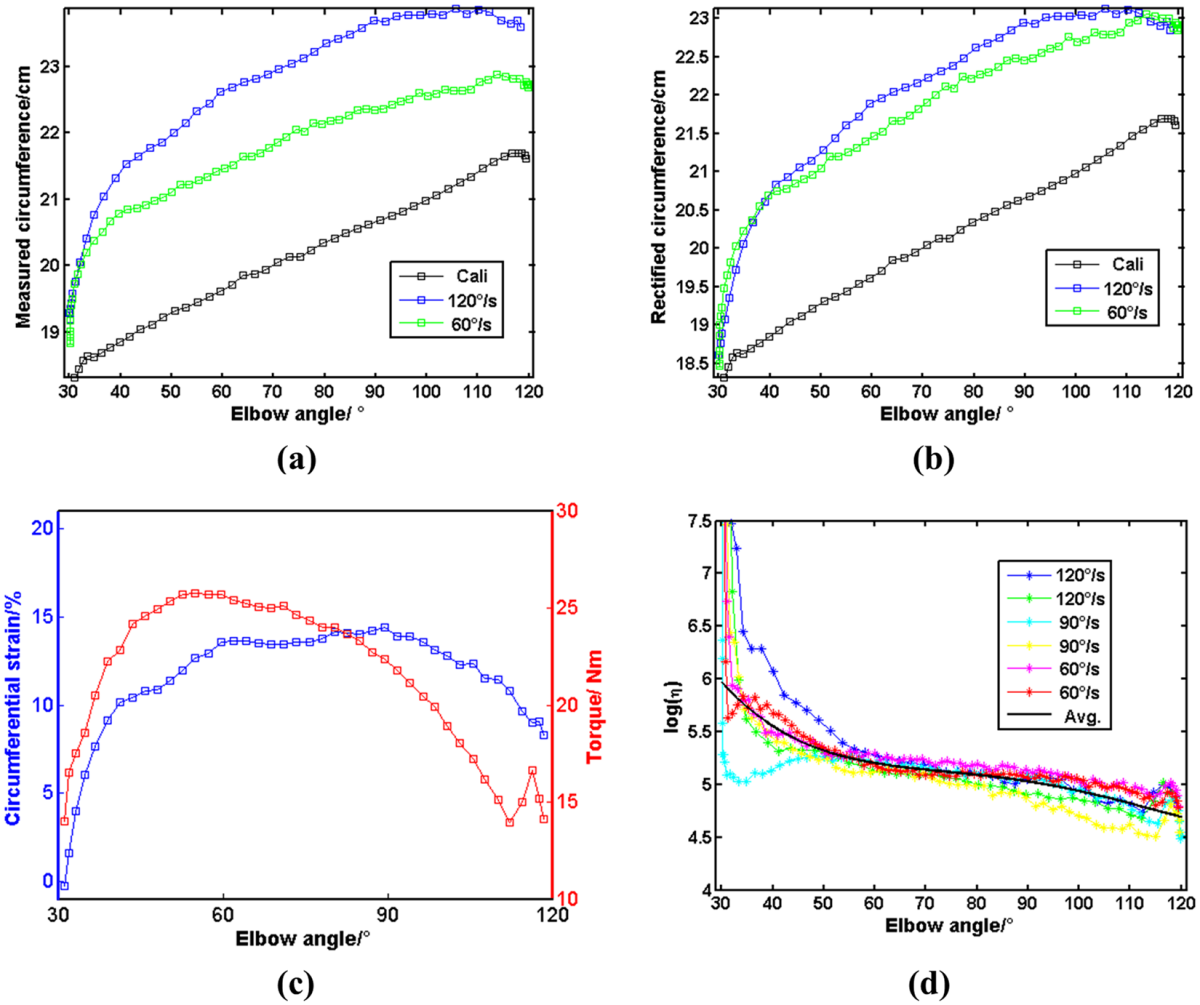
(**a**) Measured circumference; (**b**) Rectified circumference; (**c**) Typical circumferential strain and torque during isokinetic flexion (90°/s); (**d**) $\mathrm{lg}\,\eta$ in isokinetic test. All are from Subject No. 1 in isokinetic tests.

The measured upper-arm circumferences *C* were rectified (Figs 3(a) and (b)) and the circumferential strains were calculated (Fig. 3(c)) according to equations (7f) and (8), respectively. The ratio $\mathrm{lg}\,\eta$ was obtained according to equation (10) and demonstrated in Fig. 3(d). For same subject, profiles of the $\mathrm{lg}\,\eta$ are observed with similar trend, revealing an apparent effect of elbow angle (declining with elbow angle), which is consistent with previous findings^24^. Being approximately linear to elbow angle within ROM, $\mathrm{lg}\,\eta$ s were parameterized through linear fitting within the range of [50°, 100°]:

11a $$\mathrm{ln}\,\eta ={a}_{1}\theta +{a}_{2},\,\theta \in [{50}^{^\circ },{100}^{^\circ }]$$

where parameters *a*_1_ and *a*_2_ were obtained by least-square optimization (Supplementary Table S2, parameters obtained in linear fitting). Single-factor analysis of variation (ANOVA) was performed on the *a*_1_s and *a*_2_s to evaluate the impact of angular velocity on the $\mathrm{lg}\,\eta$s. The p-value of both the *a*_1_s and *a*_2_s were calculated and summarized in Table 1.

**Table 1:** P-values of *a*_1_s and *a*_2_s through ANOVA of lgη on angular velocity.

Subject No.	p_*a*1_	p_*a*2_
1	0.9925	0.9906
2	0.9924	0.9908
3	0.9924	0.9901
4	0.9924	0.9907
5	0.9924	0.9892
6	0.9924	0.9899
7	0.9924	0.9907
8	0.9924	0.9908
9	0.9924	0.9904
10	0.9924	0.9905
11	0.9924	0.9902
12	0.9924	0.9893
13	0.9924	0.9907

All the ${p}_{{a}_{1}}$ and ${p}_{{a}_{2}}$ obtained through the single-factor ANOVA were observed close to 1 (≫0.01), indicating there is constantly no significant difference between $\mathrm{lg}\,\eta$ s at different angular velocities (60°/s, 90°/s and 120°/s). The effect sizes for the differences among three angular velocities were also calculated, ranging from 0.065~0.070, deemed as medium-high according to Cohen^33^. Hence, the results of ANOVA are valid, which implies the effect of angular velocity is negligible in the $\mathrm{lg}\,\eta$s. This strong evidence indicates the torque-velocity factor (${G}_{v}(\dot{\theta })$) in equation (9) reduces to a constant *q*,

11b $$T=s\cdot {\mathop{G}\limits^{\frown {}}}_{l}(\theta )\cdot q$$

where it’s apparent that ${\mathop{G}\limits^{\frown {}}}_{l}(\theta )\cdot q=\eta$. This means that torque of flexions can be determined from only 2 factors, circumferential strain (*s*) and a torque-angle factor, which is largely different from the conventional Hill-based models as in equation (2c).

### Verification of the model

**Figure 4:**
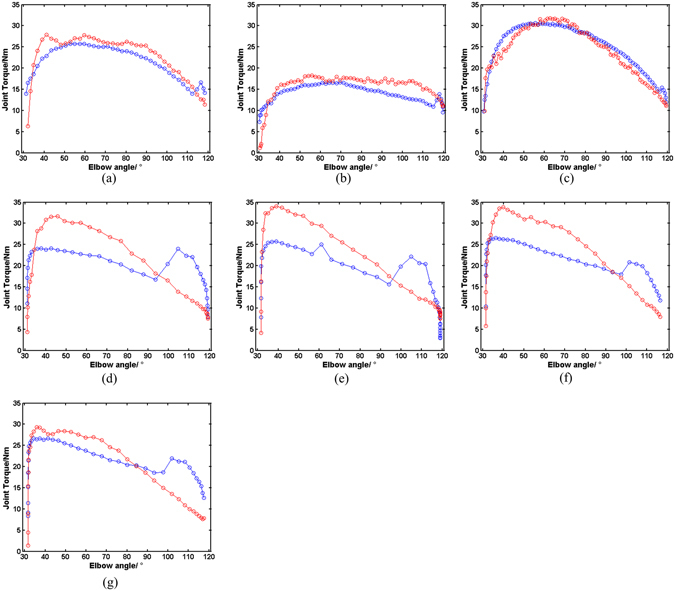
Comparison between the calculated (red) and measured torque (blue) (subject No. 1) in the 2^nd^ flexions in the 2^nd^ elbow kinetic flexion at: (**a**) 90°/s, (**b**) 120°/s and (**c**) 60°/s. and that in the elbow isotonic flexions: (**d**) the 1^st^ 30% T_120_ flexion, (**e**) the 2^nd^ 30% T_120_ flexion, (**f**) the 1^st^ 40% T_120_ flexion, (**g**) the 2^nd^ 40% T_120_ flexion.

As aforementioned, subjects took two sets of actions (1^st^ and 2^nd^) under each angular velocity in isokinetic test. As the ratio *η* was found independent of angular velocities ($\dot{\theta }$), for each subject, it can be trained from the 1^st^ sets in isokinetic test, using average method:

11c $$\bar{\eta }(\theta )=\frac{\sum _{i=1,3,5}{\eta }_{i}}{3}$$

where *η*_1_, *η*_3_, *η*_5_ are the ratio functions obtained in 1^st^ set of actions at 60°/s, 90°/s and 120°/s, respectively. Once the $\bar{\eta }$ is determined, torque can be calculated from circumferential strain in any kinetic elbow flexions. For verification of the model, torque was estimated for the 2^nd^ sets of isokinetic flexions, as well as for all the isotonic flexions, based on equation (11b). Comparisons between the estimated torque from the model and measured torque by Biodex 3 were demonstrated in Fig. 4 (demoed with subject No. 1).

Error of calculated torque was evaluated using 5 indicators: the correlation coefficient (*C*.*C*), the maximum absolute error (*r*_*ma*_), the maximum relative error (*r*_*mr*_), the root mean square error (*r*_*rms*_), and the mean relative error (*r*_*m*_). Evaluation was only limited to the range of 50°~100°, which is the stable and effective range of Biodex 3 in isotonic mode, avoiding the unstable torque in both starting and ending of elbow flexions.

11d $$\begin{array}{ccc}C.C & = & \frac{conv({T}_{m},{T}_{c})}{\sigma ({T}_{m})\cdot \sigma ({T}_{c})}\\ {r}_{ma} & = & \max |{T}_{m}-{T}_{c}|;\,{r}_{mr}=\,\max |\frac{{T}_{m}-{T}_{c}}{{T}_{m}}|\\ {r}_{rms} & = & \sqrt{\frac{\sum _{i=1-N}{|{T}_{mi}-{T}_{ci}|}^{2}}{N}};\,{r}_{m}=\frac{{r}_{rms}}{{\bar{T}}_{m}}\end{array}$$

where *T*_*m*_ and *T*_*c*_ are the measured and calculated torque based on the present model, respectively. The errors of torque estimation for the thirteen subjects were summarized in Table S3 (Supplementary Table S3, evaluation of the errors in isokinetic and isotonic flexions), of which *r*_*rms*_ and *r*_*m*_ are listed in Table 2.

**Table 2:** Errors of estimation (*r*_*rms*_ and *r*_*m*_) in isokinetic and isotonic flexions.

Subject No	Isokinetic		Isotonic	
	*r*_*rms*_(N•m)	*r*_*m*_	*r*_*rms*_(N•m)	*r*_*m*_
1	2.0964	0.0500	5.8193	0.2133
2	6.1742	0.2061	6.3443	0.1812
3	6.8885	0.1281	7.8759	0.2131
4	5.6927	0.3020	9.1720	0.4013
5	6.1465	0.1333	6.4430	0.2290
6	11.9803	0.2900	6.4530	0.2195
7	2.8041	0.0620	7.8748	0.3089
8	3.6302	0.0691	4.0440	0.1057
9	5.3530	0.2127	3.6301	0.1164
10	6.2079	0.0852	7.7643	0.2344
11	4.5536	0.1172	6.4729	0.2893
12	6.516	0.2145	7.3968	0.2565
13	5.2001	0.1029	6.7100	0.1427
Mean ± Std	5.6341 ± 2.3120	0.1518 ± 0.0815	6.6154 ± 1.4665	0.2239 ± 0.0776

Mean relative error (*r*_*m*_) of most estimations were lower than 30%, except for subject No. 4, with a *r*_*m*_ of 30% in isokinetic and 40% in isotonic flexions, namely the worst estimation. The best estimation was with subject No. 8, with a mean relative error of 7% in isokinetic and 11% in isotonic flexions. The averaged *r*_*m*_ is 15% and 22% in isokinetic and isotonic flexions, respectively. Generally, the error of estimated torque in isokinetic is smaller than in isotonic flexions.

## Discussions and Limitations

The conditions under which the model derivation is valid shall be discussed first. Two elbow flexors (biceps brachii and brachioradialis) and triceps brachii all contribute to the circumferences’ variation during elbow flexions. Although maximum voluntary elbow flexions were performed by subjects, triceps brachii would inevitably co-contract, which has been totally ignored. Secondly, compression garments with safe pressures on human (under 30 mm Hg) have been reported as not hindering skeletal muscles’ deformation and undermining muscle contraction^34, 35^. As soft tissue, biceps brachii can be squashed by the sensing belt of LGMS, leading to underestimated measured circumferences. To which extent this pressure affects the accuracy of circumferences has not been addressed. Thirdly, as dominant elbow flexors, biceps brachii is a parallel-fibered skeletal muscle (with fibers parallel to the force-generating axis). Hence, any increment of fiber’s diameter would directly lead to an increase in muscle thickness and upper-arm circumference, which automatically establish the feasibility of this work. For pennated skeletal muscles, however, the increment of thickness is only a radial component of increase in fiber diameters. Moreover, pennation angle also varies during contraction^10, 11, 36^. It’s then complicated to utilize limb circumference for monitoring contraction. Finally, only the loading phases (flexions, when biceps contract) has been analyzed, similar to most of published works focusing on the loading phase of skeletal muscles instead of relaxation^14, 22, 30, 37^. This selection was to ensure the fabric strain sensors being incrementally stretched to control the error due to relaxation.

Supported by the experimentally derived factors of muscle length and velocity, the model exhibits much less error than previous related work^22^. The new model seems to work better for isokinetic flexions (15% error) than isotonic flexions (22% error), which is natural, since the model itself is determined in isokinetic modes. Meanwhile, subjects might unconsciously relocate elbow axis in isotonic flexions, which were generally more rapid compared to isokinetic, lowering the measured torque. Besides, minus correlation coefficients were observed between estimated and measured torque (such as −0.1718 for subject No. 6 in isotonic flexions, Table S3). The reason is that in some cases, the estimated torque was close to measured torque but with an opposite trend within a small inner range, leading to a minus correlation coefficient. Currently, the model has not been statistically verified through statistical hypothesis testing. The reason is that, first, it’s been observed that over-estimation of torque only happened in isotonic flexion, indicating that unknown factors particular in isotonic flexions may be taking effect. Whether or not the factors are in the model would be determined in future. Also, there is a lack of repeated trials. Currently, this work was supported by only thirteen subjects with thirteen sets of data. A further confirmation of the model with more subjects enrolled would be marked in future, as well as a statistical analysis of error to evaluate the repeatability of the model.

It is a very important finding of the current study that the velocity does not affect the relationship between the torque and circumferential strain. In other word, *G*_*v*_ is a constant independent of velocity in equation (11b). The possible explanations are as follows. The velocity factor *G*_*v*_ is a product of *k*_2_ and *g*_*v*_. The former is related to the velocity factor determined by the circumference strain data obtained from LGMS, and the latter is linked with the velocity factor determined from sEMG data during muscle contraction. If the two factors were inversely proportional to each other, then it is possible that the combined effect of *g*_*v*_ and *k*_2_ makes their product independent of the velocity while individual ones still hold their velocity related relationship. According to Huxley’s sliding filament theory^38^, the attaching-detaching process of cross-bridges between myosins and actins depends on muscle length and shortening velocity. This means that the maximum contraction of skeletal muscle depends on length and velocity, which is consistent with Hill’s findings. However, the tension of muscle depends only on the numbers of functional cross-bridges, when myosin turns its head to climb on actin. Muscle thickness only increases when the tight myosin-actin structure shortens and expands sarcomere’s diameter, which further leads to increments in limb circumference. The sEMG and LGMS may record the different but related processes of muscle contraction and thickening, respectively. This phenomenon requires further detailed investigation by comparative study of sEMG and LGMS.

## Conclusions

A new bio-mechanical model for elbow kinetic flexions has been derived, linking joint torque with circumferential strain. The ratio *η* (combination of torque-angle and torque-velocity effect) has been examined by single-factor ANOVA on three angular velocities (90°/s, 120°/s and 60°/s) and for thirteen subjects. It has been found that for all the subjects, the change of velocity has no effect on the ratio. Hence, the torque-velocity factor reduced to constant and the new model was largely simplified. Verification of the model was conducted using elbow isokinetic and isotonic flexions. Comparisons were made between the torque calculated from the model and that measured by Biodex^®^ 3. The model was verified satisfactorily since the averaged mean relative error was observed as 15% and 22% in isokinetic and isotonic flexions, respectively.

In summary, when using upper-arm circumferential strain to monitoring contraction, generated torque of elbow flexion depends on only 3 factors, maximum torque, circumferential strain and the torque-length factor, compared to the 4 factors when using sEMG. This finding opens doors for further exciting applications of smart wearable technologies on monitoring skeletal muscles’ contraction. Moreover, since circumferential measurements are based on the changes in morphological parameters of skeletal muscles, the bio-mechanical study of the elbow-muscular system provides a solid scientific ground for the data interpretation.

## Electronic supplementary material

Supplementary information

## References

[1] Hill AV (1938). The Heat of Shortening and the Dynamic Constants of Muscle. Proceedings of the Royal Society B: Biological Sciences.

[2] Bigland B, Lippold OCJ (1954). The relation between force, velocity and integrated electrical activity in human muscles. The Journal of physiology.

[3] Winters, J. M. In Multiple muscle systems Hill-based muscle models: a systems engineering perspective Ch. 5, 69–93 (Springer, 1990).

[4] Ramsay JW, Hunter BV, Gonzalez RV (2009). Muscle moment arm and normalized moment contributions as reference data for musculoskeletal elbow and wrist joint models. J Biomech.

[5] Rahikainen A, Avela J, Virmavirta M (2012). Modeling the Force-Velocity Relationship in Arm. World Journal of Mechanics.

[6] Zajac FE (1992). How Musculotendon Architecture and Joint Geometry Affect the Capacity of Muscles to Move and Exert Force on Objects - a Review with Application to Arm and Forearm Tendon Transfer Design. Journal of Hand Surgery-American Volume.

[7] Biewener AA, Wakeling JM, Lee SS, Arnold AS (2014). Validation of Hill-Type Muscle Models in Relation to Neuromuscular Recruitment and Force-Velocity Properties: Predicting Patterns of *In Vivo* Muscle Force. Integrative and Comparative Biology.

[8] Cao H, Boudaoud S, Marin F, Marque C (2015). Surface EMG-force modelling for the biceps brachii and its experimental evaluation during isometric isotonic contractions. Comput Methods Biomech Biomed Engin.

[9] Franchi MV (2014). Architectural, functional and molecular responses to concentric and eccentric loading in human skeletal muscle. Acta Physiol (Oxf).

[10] Kwah LK, Pinto RZ, Diong J, Herbert RD (2013). Reliability and validity of ultrasound measurements of muscle fascicle length and pennation in humans: a systematic review. J Appl Physiol.

[11] Strasser EM, Draskovits T, Praschak M, Quittan M, Graf A (2013). Association between ultrasound measurements of muscle thickness, pennation angle, echogenicity and skeletal muscle strength in the elderly. Age.

[12] Akagi R (2015). Determination of Contraction-Induced Changes in Elbow Flexor Cross-Sectional Area for Evaluating Muscle Size-Strength Relationship during Contraction. J Strength Cond Res.

[13] Sitilertpisan P (2011). Comparison of lateral abdominal muscle thickness between weightlifters and matched controls. Phys Ther Sport.

[14] Guo JY, Zheng YP, Xie HB, Chen X (2010). Continuous monitoring of electromyography (EMG), mechanomyography (MMG), sonomyography (SMG) and torque output during ramp and step isometric contractions. Med Eng Phys.

[15] Starkey DB (1996). Effect of resistance training volume on strength and muscle thickness. Med Sci Sports Exerc.

[16] Tresignie J, Scafoglieri A, Cattrysse E, Clarys JP (2012). Cross-sectional content analysis of clinically applied circumferences. European Journal of Clinical Investigation.

[17] Hodges PW, Pengel LH, Herbert RD, Gandevia SC (2003). Measurement of muscle contraction with ultrasound imaging. Muscle & nerve.

[18] Abe T, Loenneke JP, Thiebaud RS (2015). Morphological and functional relationships with ultrasound measured muscle thickness of the lower extremity: a brief review. Ultrasound.

[19] Akagi R (2014). Association Between Contraction-Induced Increases in Elbow Flexor Muscle Thickness and Distal Biceps Brachii Tendon Moment Arm Depends on the Muscle Thickness Measurement Site. Journal of Applied Biomechanics.

[20] Shi J, Zheng YP, Huang QH, Chen X (2008). Continuous monitoring of sonomyography, electromyography and torque generated by normal upper arm muscles during isometric contraction: Sonomyography assessment for arm muscles. Ieee T Bio-Med Eng.

[21] Cameron N (2013). Essential anthropometry: Baseline anthropometric methods for human biologists in laboratory and field situations. Am J Hum Biol.

[22] Kim WS (2014). Development of a muscle circumference sensor to estimate torque of the human elbow joint. Sensors and Actuators A: Physical.

[23] Cannan JAR, Hu HS (2011). Automatic Circumference Measurement for Aiding in the Estimation of Maximum Voluntary Contraction (MVC) in EMG Systems. Intelligent Robotics and Applications, Pt I.

[24] Wang, X. *et al*. Monitoring elbow isometric contraction by novel wearable fabric sensing device. *Smart Mater Struct* **25**, doi:Artn 12502210.1088/0964-1726/25/12/125022 (2016).

[25] Bouillard K, Nordez A, Hodges PW, Cornu C, Hug F (2012). Evidence of changes in load sharing during isometric elbow flexion with ramped torque. J Biomech.

[26] Gennisson JL, Cornu C, Catheline S, Fink M, Portero P (2005). Human muscle hardness assessment during incremental isometric contraction using transient elastography. J Biomech.

[27] Buchanan TS, Lloyd DG, Manal K, Besier TF (2005). Estimation of muscle forces and joint moments using a forward-inverse dynamics model. Med Sci Sports Exerc.

[28] Tsai LC, Colletti PM, Powers CM (2012). Magnetic Resonance Imaging-Measured Muscle Parameters Improved Knee Moment Prediction of an EMG-Driven Model. Med Sci Sports Exerc.

[29] Brown, I. E., Liinamaa, T. L. & Loeb, G. E. Relationships between range of motion, L(0), and passive force in five strap-like muscles of the feline hind limb. *J Morphol* **230**, 69–77, doi:10.1002/(Sici)1097-4687(199610)230:169::Aid-Jmor63.0.Co;2-I (1996). 10.1002/(SICI)1097-4687(199610)230:1<69::AID-JMOR6>3.0.CO;2-I 8843689

[30] Campy RM, Coelho AJ, Pincivero DM (2009). EMG-torque relationship and reliability of the medial and lateral hamstring muscles. Med Sci Sports Exerc.

[31] Wang YY (2011). Novel fabric pressure sensors: design, fabrication, and characterization. Smart Mater Struct.

[32] Yi WJ, Wang YY, Wang GF, Tao XM (2012). Investigation of carbon black/silicone elastomer/dimethylsilicone oil composites for flexible strain sensors. Polymer Testing.

[33] Cohen, J. Statistical power analysis for the behavioral sciences (revised ed.). (New York: Academic Press, 1977).

[34] Born DP, Sperlich B, Holmberg HC (2013). Bringing Light Into the Dark: Effects of Compression Clothing on Performance and Recovery. International Journal of Sports Physiology and Performance.

[35] Pereira MC (2014). The Effects of Graduated Compression Sleeves on Muscle Performance: A Randomised Controlled Trial. International Journal of Sports Science & Coaching.

[36] Fukunaga T, Ichinose Y, Ito M, Kawakami Y, Fukashiro S (1997). Determination of fascicle length and pennation in a contracting human muscle *in vivo*. J Appl Physiol.

[37] Clancy EA, Bida O, Rancourt D (2006). Influence of advanced electromyogram (EMG) amplitude processors on EMG-to-torque estimation during constant-posture, force-varying contractions. J Biomech.

[38] Huxley AF, Niedergerke R (1954). Structural changes in muscle during contraction; interference microscopy of living muscle fibres. Nature.

