# Supplementary material for: A Bio-mechanical Model for Elbow Isokinetic and Isotonic Flexions
Source: Sci Rep. 2017 Aug 21;7:8919. doi: 10.1038/s41598-017-09071-x (PMC5567174; doi:10.1038/s41598-017-09071-x)
Supplement: Supplementary file 1 — Supplementary information [file 41598_2017_9071_MOESM1_ESM.pdf]

Supplementary Information for:

A Bio-mechanical Model for Elbow Isokinetic and Isotonic Flexions

Xi Wang [1], Xiaoming Tao [1,2], Raymond C.H. So [3]

[1] Institute of Textiles and Clothing, The Hong Kong Polytechnic University, Hong Kong, China
[2] Multidisciplinary Division of Bioengineering, The Hong Kong Polytechnic University, Hong Kong, China
[3] Hong Kong Sports Institute, Hong Kong, China

Supplementary **Table S1** Physical information of subjects

Subjects No.	Age	Gender	Height (cm)	Weight (kg)	BMI	Dominant Hand	Upper-arm Circum. (extended and relaxed) (cm)
1	33	M	165.0	65.0	23.9	R	30.0
2	22	M	173.0	57.0	19.0	R	26.5
3	20	M	173.5	67.3	22.4	R	27.0
4	33	M	170.1	71.8	24.8	R	31.5
5	29	M	169.0	77.0	27.0	R	30.0
6	33	M	180.0	72.8	22.5	R	30.5
7	26	M	178.0	58.0	18.3	R	26.5
8	23	M	175.0	80.0	26.1	R	30.0
9	28	M	175.4	63.0	20.5	R	25.5
10	30	M	175.0	82.0	26.8	R	31.5
11	25	M	176.0	70.0	22.6	R	27.0
12	26	M	170.7	62.5	21.4	R	26.5

13	31	M	178.0	70.0	22.1	R	29.0

Supplementary **Table S2** a_1 and a_2 obtained through linear fitting (Subject No.1)

Angular velocity (˚/s)	a_1	a_2
120	-0.0171	4.4177
120	-0.0146	4.3631
90	-0.0138	4.3389
90	-0.0177	4.4003
60	-0.0114	4.2412
60	-0.0111	4.2005

Supplementary **Table S3** Evaluation of the errors in (a) isokinetic and (b) isotonic flexions

(a)

Subject No	*C.C*	r_{ma} (Nm)	r_{rms} (Nm)	r_{mr}	r_m
1	0.5577	1.1195	2.0964	0.1028	0.0500
2	0.4335	3.4153	6.1742	0.3891	0.2061
3	-0.0234	3.6269	6.8885	0.2437	0.1281
4	0.9916	4.3514	5.6927	0.6571	0.3020
5	0.9679	3.5052	6.1465	0.3824	0.1333
6	0.0010	7.0816	11.9803	0.5726	0.2900
7	0.7935	1.6098	2.8041	0.1114	0.0620
8	0.2585	2.2611	3.6302	0.1088	0.0691
9	-0.0922	3.2568	5.3530	0.3843	0.2127
10	0.6288	3.1610	6.2079	0.1623	0.0852
11	-0.1336	2.9907	4.5536	0.1774	0.1172
12	0.6693	4.5047	6.516	0.3162	0.2145
13	0.6897	3.4035	5.2001	0.1534	0.1029
Mean	0.4417	3.4067	5.6341	0.2893	0.1518

(b)

Subject No	*C.C*	r_{ma} (Nm)	r_{rms} (Nm)	r_{mr}	r_m
1	0.9821	4.4403	5.8193	0.258	0.2133

2	0.8301	3.1443	6.3443	0.3637	0.1812
3	0.8181	4.7153	7.8759	0.3387	0.2131
4	0.9863	7.2956	9.1720	0.6293	0.4013
5	0.9569	4.5507	6.4430	0.2943	0.2290
6	-0.1718	4.0167	6.4530	0.4116	0.2195
7	0.0349	5.8610	7.8748	0.4354	0.3089
8	-0.0567	2.5729	4.0440	0.1610	0.1057
9	-0.0025	2.0725	3.6301	0.2165	0.1164
10	0.8107	5.4254	7.7643	0.3236	0.2344
11	0.5915	5.0883	6.4729	0.3959	0.2893
12	-0.8266	4.8353	7.3968	0.3903	0.2565
13	0.6644	4.0026	6.7100	0.2343	0.1427
Mean	0.4321	4.4631	6.6154	0.3425	0.2239
